# Early Intervention Programmes in Psychosis

**DOI:** 10.1192/j.eurpsy.2022.38

**Published:** 2022-09-01

**Authors:** M. Nordentoft

**Affiliations:** CORE-Copenhagen Research Center for Mental Health, Mental Health Center Copenhagen, Hellerup, Denmark

## Abstract

Early Intervention Programmes in Psychosis Mental illness is associated with high burden of disease and it has severe individual and societal consequences. In first episode psychosis, specialised Early Intervention Services with team-based intensive case management and family involvement are superior to standard treatment in reducing psychotic and negative symptoms and comorbid substance abuse and improving social functioning and user satisfaction. The results of the OPUS-trials will be presented together with meta-analyses based on similar trials. The sustainability of positive effects will be highlighted with results from three different trials. Results of long-term follow-up studies indicate that the prognosis of first episode psychosis is very diverse with the extremes represented by one group being well functioning and able to quit medication without relapse; and another group having a long-term chronic course of illness with a need for support to maintain daily activities. The latter is, in spite of many efforts, still poorly served. A substantial proportion of patients with psychosis have treatment resistant auditory hallucinations. The UK-based AVATAR-trial was the first to demonstrate effect of simulation training on devaluating voices. The Danish CHALLENGE-trial aim to replicate the findings from the AVATAR trial in a virtual reality setting.

**Disclosure:**

No significant relationships.

